# Macrophages play a leading role in determining the direction of astrocytic migration in spinal cord injury via ADP-P2Y1R axis

**DOI:** 10.1038/s41598-023-38301-8

**Published:** 2023-07-10

**Authors:** Gentaro Ono, Kazu Kobayakawa, Hirokazu Saiwai, Tetsuya Tamaru, Hirotaka Iura, Yohei Haruta, Kazuki Kitade, Keiichiro Iida, Kenichi Kawaguchi, Yoshihiro Matsumoto, Makoto Tsuda, Tomohiko Tamura, Keiko Ozato, Kazuhide Inoue, Dai-Jiro Konno, Takeshi Maeda, Seiji Okada, Yasuharu Nakashima

**Affiliations:** 1 Department of Orthopaedic Surgery, Graduate School of Medical Sciences, Kyushu University, 3-1-1, Maidashi, Higashi-ku, Fukuoka, 812-8582 Japan; 2 Department of Molecular and System Pharmacology, Graduate School of Pharmaceutical Sciences, Kyushu University, 3-1-1 Maidashi, Higashi-ku, Fukuoka, 812-8582 Japan; 3 Kyushu University Institute for Advanced Study, Kyushu University, 744 Motooka, Nishi-ku, Fukuoka-shi, Fukuoka 819-0395 Japan; 4 Department of Immunology, Yokohama City University Graduate School of Medicine, 3-9 Fukuura, Kanazawa-ku, Yokohama 236-0004 Japan; 5 Program in Genomics of Differentiation, Section on Molecular Genetics of Immunity, Division of Developmental Biology, NICHD, National Institutes of Health, Building 6A, Room 2A01, 6 Center Drive, Bethesda, MD 20892 USA; 6 Greenpharma Research Center for System Drug Discovery, Kyushu University, 3-1-1 Maidashi, Higashi-ku, Fukuoka, 812-8582 Japan; 7 Department of Energy and Materials, Faculty of Science and Engineering, Kindai University, Osaka, 577-8502 Japan; 8 Department of Orthopaedic Surgery, Spinal Injuries Center, 550-4 Igisu, Iizuka, Fukuoka 820-8508 Japan; 9 Department of Orthopaedic Surgery, Graduate School of Medicine, Osaka University, 2-2 Yamada-oka, Suita, Osaka 565-0871 Japan

**Keywords:** Neuroscience, Glial biology, Astrocyte, Cell migration

## Abstract

After spinal cord injury (SCI), inflammatory cells such as macrophages infiltrate the injured area, and astrocytes migrate, forming a glial scar around macrophages. The glial scar inhibits axonal regeneration, resulting in significant permanent disability. However, the mechanism through which glial scar-forming astrocytes migrate to the injury site has not been clarified. Here we show that migrating macrophages attract reactive astrocytes toward the center of the lesion after SCI. Chimeric mice with bone marrow lacking IRF8, which controls macrophage centripetal migration after SCI, showed widely scattered macrophages in the injured spinal cord with the formation of a huge glial scar around the macrophages. To determine whether astrocytes or macrophages play a leading role in determining the directions of migration, we generated chimeric mice with reactive astrocyte-specific *Socs3*^−/−^ mice, which showed enhanced astrocyte migration, and bone marrow from *IRF8*^−/−^ mice. In this mouse model, macrophages were widely scattered, and a huge glial scar was formed around the macrophages as in wild-type mice that were transplanted with *IRF8*^−/−^ bone marrow. In addition, we revealed that macrophage-secreted ATP-derived ADP attracts astrocytes via the P2Y1 receptor. Our findings revealed a mechanism through which migrating macrophages attract astrocytes and affect the pathophysiology and outcome after SCI.

## Introduction

Although spinal cord injury (SCI) causes severe disability, effective treatments have not been established^1^. When the blood-spinal cord barrier is disrupted by mechanical injury, inflammatory cells, such as neutrophils and macrophages, infiltrate into the spinal cord, and these cells secrete inflammatory cytokines, such as interleukin (IL)-1α, IL-1β, IL-6, and tumor necrosis factor (TNF)-α, causing secondary damage^2^. The influx of inflammatory cells activates resident glia, including astrocytes, and remodels the extracellular matrix with an increase in fibronectin, collagen, and laminin^3^. The extracellular matrix and inflammatory cells are located at the center of the injury, and astrocytes migrate toward them to form a glial scar that serves as a physical barrier. Since this barrier prevents axonal regeneration, elucidation of the mechanism of glial scar formation is critical for developing new treatments for SCI.

We previously reported that Stat3 regulates astrocyte migration^4^. However, the factors that attract astrocytes to the injury site remain unknown, although astrocyte migration is one of the most important processes in glial scar formation. In this study, we attempted to identify factors that attract astrocytes to the injury center by focusing on macrophages inside the glial scar. The proportion of macrophages at the injury center varies over time. Especially after the subacute stage, when the glial scar is formed, the infiltration of macrophages is particularly pronounced (7 days post-injury)^5^, suggesting that macrophages may also be involved in the formation of the glial scar. We hypothesized that macrophages might also be involved in the appearance of glial scars. We reported that in *IRF8*^−/−^ mice, in which macrophages are widely scattered after SCI due to impaired migration with the downregulated expression of purinergic receptors, larger glial scars were formed in comparison to wild-type (WT) mice^6^. In the central nervous system (CNS), astrocytes form close intercellular communication with other cell types, such as neurons and microglia, in which purine receptors play a significant role, for example, in regulating homeostasis, with consequences for synaptic transmission, and higher-order cognitive processes^7,8^. In addition, macrophages have been shown to attract other macrophages by secreting ATP, the ligand for the purine receptor^9^. Therefore, we hypothesized that macrophages also induce astrocytes via purine receptors. In this study, we demonstrated the effect of macrophages on astrocyte migration after SCI, which is critical for glial scar formation.

## Results

### Macrophages attract astrocytes in SCI

**Figure 1:**
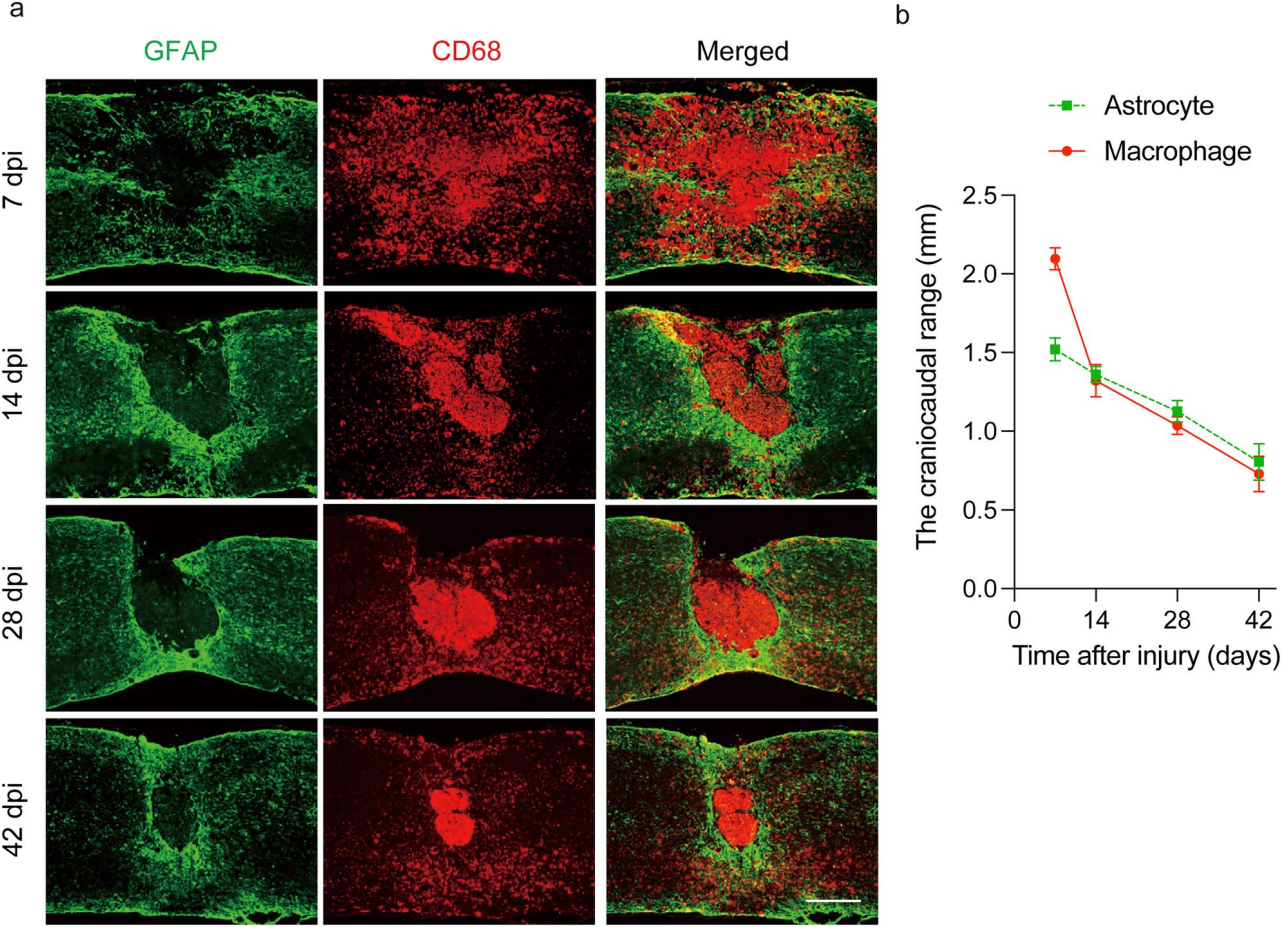
Temporal changes in macrophages and astrocytes in SCI. (**a**) Macrophage migration occurred after SCI, followed by glial scar formation by astrocytes at 7–14 days post-injury (dpi). Scale bar: 500 μm. (**b**) Quantitative analysis of the craniocaudal range of astrocytes in the glial scar and macrophages (n = 6 per group). Error bars indicate the SEM.

**Figure 2:**
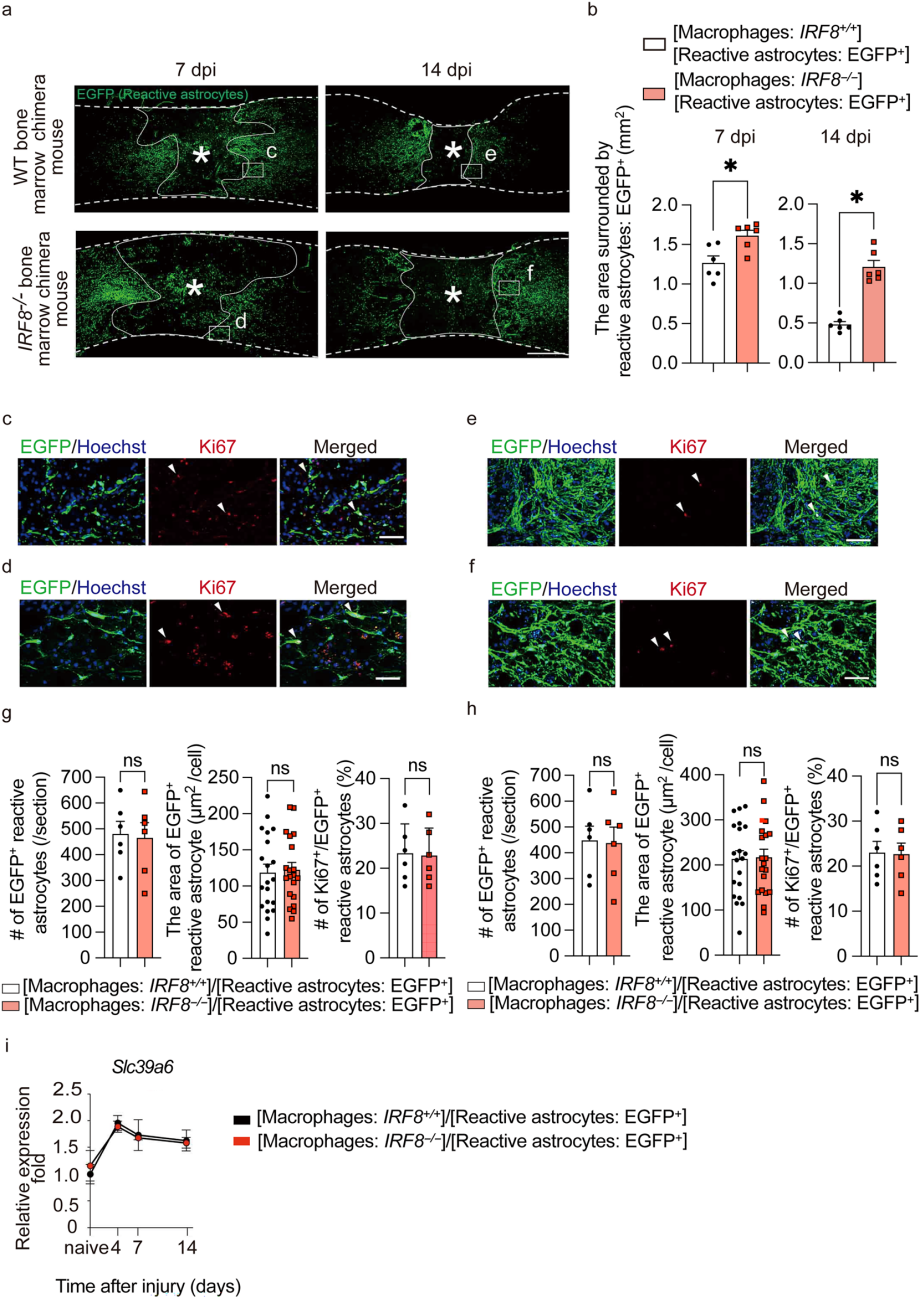
Inhibition of macrophage migration results in larger glial scars. (**a**) Change in the distribution of EGFP^+^ reactive astrocytes over time in the injured spinal cord of [reactive astrocytes: *Nes*-*Cre*-*EGFP*^+^/macrophages: WT] and [reactive astrocytes: *Nes*-*Cre*-*EGFP*^+^/macrophages: *IRF8*^−/−^] mice. Reactive astrocytes: *Nes*-*Cre*-*EGFP*^+^/macrophages: *IRF8*^−/−^ mice had larger glial scars. Scale bar: 500 μm. (**b**) Quantitative analysis of the area surrounded by EGFP-positive cells: reactive astrocytes. There were significant differences between [reactive astrocytes: *Nes*-*Cre*-*Socs3*^−/−^*EGFP*^+^/macrophages: WT] and [reactive astrocytes: *Nes*-*Cre*-*Socs3*^−/−^*EGFP*^+^/macrophages: IRF8^−/−^] mice at 7 days and 14 dpi (n = 6 per group at each time point). (**c**) High-magnification view of WT bone chimeric mice at 1 week after injury. Scale bar: 100 μm. (**d**) High-magnification view of *IRF8*^*−/−*^ bone chimeric mice at 1 week after injury. Scale bar: 100 μm. (**e**) High-magnification view of WT bone chimeric mice at 2 weeks after injury. Scale bar: 100 μm. (**f**) High-magnification view of *IRF8*^*−/−*^ bone chimeric mice 2 weeks after injury. Scale bar: 100 μm. (**g**) Quantitative analysis of the number of reactive astrocytes, the area of astrocyte cell bodies, and a proliferation assessment by Ki67 staining at 7 dpi. No significant differences were found in any of the parameters (n = 6 per group). (**h**) Quantitative analysis of the number of reactive astrocytes, the area of astrocyte cell bodies, and a proliferation assessment by Ki67 staining at 14 dpi. No significant differences were found in any of the parameters (n = 6 per group). (**i**) The time course of *Slc39a6* expression in the injured spinal cord determined by real-time RT‒PCR in [reactive astrocytes: *Nes-Cre-Socs3*^*−/−*^*EGFP*^+^/macrophages: WT] and [reactive astrocytes: *Nes*-*Cre*-*Socs3*^−/−^*EGFP*^+^/macrophages: *IRF8*^*−/−*^] mice (n = 6 per group at each time point). Each group was normalized to *Gapdh* values. There were no significant differences between [reactive astrocytes: *Nes-Cre-Soc3*^*−/−*^*EGFP*^+^/macrophages: WT] and [reactive astrocytes: *Nes*-*Cre*-*Socs3*^−/−^*EGFP*^+^/macrophages: *IRF8*^*−/−*^] mice. *p < 0.05, unpaired t-test. Error bars indicate the SEM.

After SCI, macrophages infiltrate into the injury site and migrate toward the epicenter with a shift in reactive astrocyte distribution toward the peri-injury area^4,6^. Initially, the distribution of scattered macrophages and astrocytes shifts toward the epicenter over time as well, but eventually, they are located in different sites, with macrophages and microglia in the center of the lesion and astrocytes at the margin of the injury (Fig. 1a,b). These results suggest that macrophage migration may affect the shift in astrocyte distribution. To investigate what happens to astrocytes when macrophage migration is impaired, a bone marrow chimera mouse model was created by transplanting bone marrow from *IRF8*^*−/−*^ mice (which have been reported to show impaired macrophage migration^6^) into *Nes-Cre-EGFP* mice, which showed the expression of EGFP in reactive astrocytes (Supplementary Fig. S1a,b). Thus, in this chimeric mouse, the function of astrocytes was normal, and only macrophages showed an impaired migration ability due to the knockout of *IRF8*. Using the chimeric mouse models, we compared the scar area at 7 and 14 days after SCI and found that the glial scar area in mice with impaired macrophage migration was significantly greater than that in mice with normal macrophage migration at both time points (Fig. 2a,b). These results suggest that the migration of macrophages influences the shift in reactive astrocyte distribution and subsequent astrocytic scar formation. In addition, we investigated whether the intrinsic nature of astrocytes was altered with impairment of macrophage migration. First, since astrocytes are known to overlap processes at the edge of the injury area, resulting in scar formation, we evaluated the effect on overlapping processes via cell density. We found no significant difference in cell density at either 7 or 14 days post-injury, indicating that loss of *IRF8* in macrophages had no significant effect on the overlapping of processes by astrocytes (Fig. 2c–h). Next, since activated astrocytes are hypertrophied, we investigated whether astrocyte activity was affected by assessing the area of astrocyte cell bodies. As a result, the area of EGFP^+^ astrocytes was not altered by impaired macrophage migration, suggesting that impaired macrophage migration had no significant effect on the overlapping of processes by astrocytes (Fig. 2c–h). Finally, the proliferative capacity of astrocytes was evaluated by Ki67 staining. No significant differences were found in proliferation between astrocytes in these two chimeric mice (Fig. 2c–h). These findings suggest that macrophages attract astrocytes extrinsically without altering their proliferative potential. We previously reported that *Slc39a6* is involved in the Stat3-mediated migration mechanism of reactive astrocytes^4^. Therefore, we assessed the expression of *Slc39a6* and found no significant difference (Fig. 2i). This result suggests that the attraction of astrocytes by macrophages is not mediated by Stat3 in astrocytes. These findings indicate that IRF8-mediated macrophage migration affects the shift in reactive astrocyte distribution without changing the proliferation, activation, or Stat3-mediated migration capacity of reactive astrocytes.

### Macrophage migration is the leading factor in SCI

**Figure 3:**
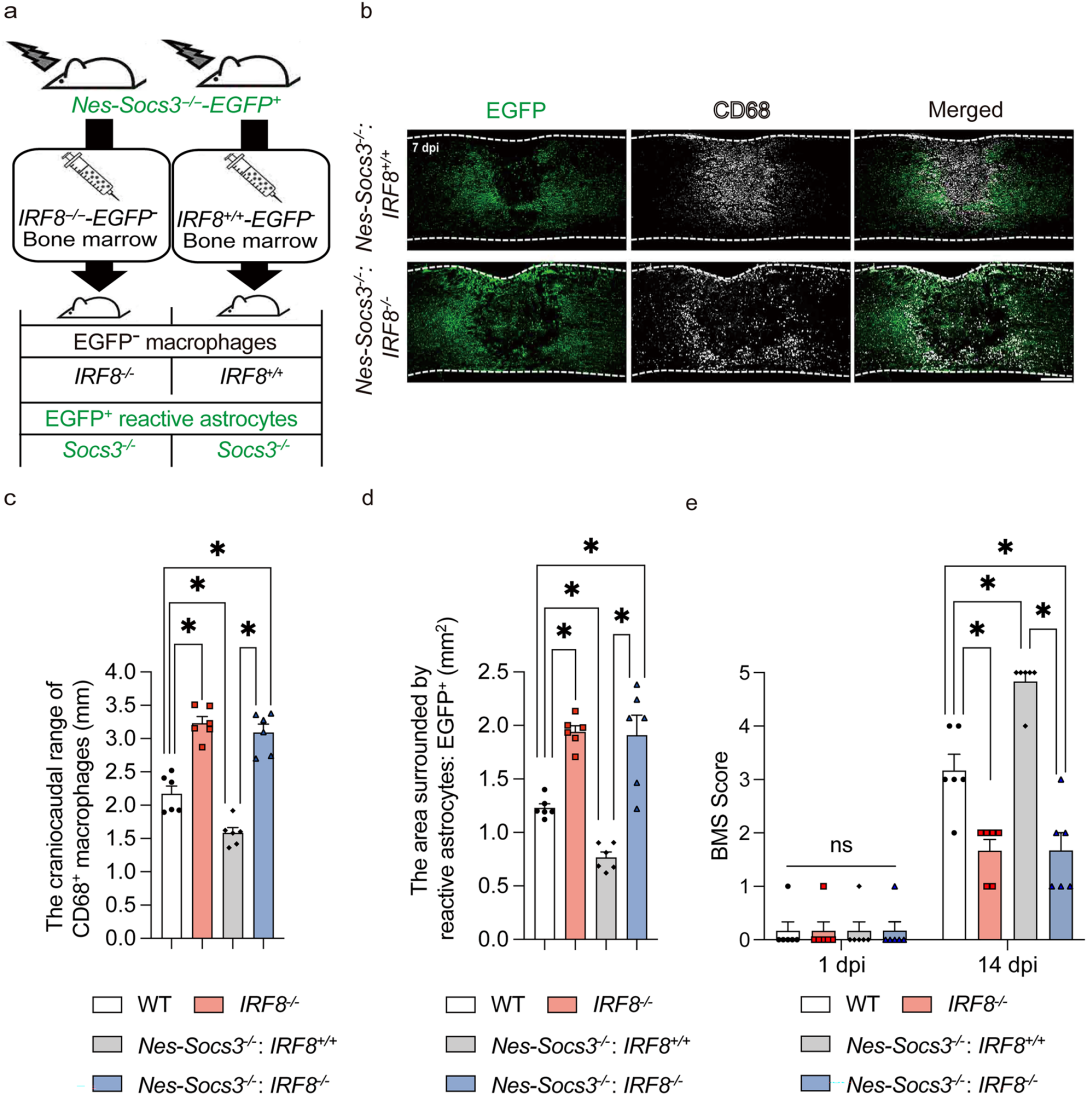
Impaired macrophage migration disturbs migration of genetically promoted migration of astrocytes after SCI. (**a**) A schematic illustration of the creation of bone marrow chimeric mice. (**b**) Immunostaining of the injured spinal cord in [reactive astrocytes: *Nes-Cre-Soc3*^*−/−*^*EGFP*^+^/macrophages: WT] and [reactive astrocytes: *Nes-Cre-Soc3*^*−/−*^*EGFP*^+^/macrophages: *IRF8*^*−/−*^] mice. Scale bar: 500 μm. (**c**) Quantitative analysis of the extent of macrophage migration. The lack of Socs3 in reactive astrocytes narrows the range of macrophage migration, while the lack of IRF8 widens the range of macrophage migration (n = 6 per group). (**d**) Quantitative analysis of the area surrounded by EGFP-positive cells: reactive astrocytes. There were significant differences in the area between [reactive astrocytes: *Nes-Cre-Soc3*^*−/−*^*EGFP*^+^/macrophages: WT] and [reactive astrocytes: *Nes-Cre-Soc3*^*−/−*^*EGFP*^+^/ macrophages: *IRF8*^*−/−*^] mice at 7 days post-injury (n = 6 per group). (**e**) The time course of motor function score after SCI. Significant differences were only seen at 14 dpi (n = 6 per group). *p < 0.05, ordinary one-way ANOVA/two-way ANOVA. Error bars indicate the SEM.

Socs3 is a negative regulator of Stat3, which is involved in the expression of intermediate filaments, cell proliferation, and cytoskeletal changes^10^. Regarding the direct involvement between astrocytic migration and these functions, it has only been reported that migration is regulated by changes in the cytoskeleton via RhoA^11^. In fact, we previously reported that astrocyte migration is enhanced and that the rapid migration of reactive astrocytes leads to the early accumulation of macrophages in *Nes-Socs3*^*−/–*^*EGFP*^+^ mice, in which the *Socs3* gene in reactive astrocytes was deleted^4^. In contrast, the poor migration of *IRF8*^*−/−*^ macrophages was accompanied by the widespread distribution of reactive astrocytes (Fig. 2a,b). These facts suggest the possibility of an interaction between infiltrating blood-derived macrophages and reactive astrocytes after SCI. To further investigate whether the migration of *IRF8*^*−/−*^ macrophages could affect the shift in reactive astrocyte distribution, we generated two additional chimeric mice: [macrophages; *IRF8*^+*/*+^]/[reactive astrocytes; *Nes-Socs3*^*−/−*^*EGFP*^+^] and [macrophages; *IRF8*^*−/−*^]/[reactive astrocytes; *Nes-Socs3*^*−/−*^*EGFP*^+^] using EGFP-negative *IRF8*^+*/*+^ or *IRF8*^*−/−*^ bone marrow cells with *Nes-Socs3*^*−/−*^*EGFP*^+^ recipient mice (Fig. 3a). In the former chimeric mice, both *IRF8*^+*/*+^ macrophages and *Nes-Socs3*^*−/−*^ reactive astrocytes showed a narrow distribution after SCI, as described in our previous study (Fig. 3b–d)^4^. However, even with *Nes-Socs3*^*−/−*^*EGFP*^+^ reactive astrocytes, the migration disorder of *IRF8*^*−/−*^ macrophages was not rescued in the latter chimeric mice (Fig. 3b–d). Instead, the *Nes-Socs3*^*−/−*^*EGFP*^+^ reactive astrocytes with *IRF8*^*−/−*^ macrophages were more widely distributed than those with *IRF8*^+*/*+^ macrophages (Fig. 3b,d). These results may indicate that while the migration of macrophages and reactive astrocytes interact with one another, the migration of macrophages is more strongly affected by macrophage IRF8 than by the enhancement of reactive astrocyte migration. We further examined the interaction between the migration of macrophages and reactive astrocytes on functional recovery after SCI. There was no significant difference among WT, *IRF8*^*−/−*^ and these chimeric mice before SCI or at 1 day post-injury (Fig. 3e). However, at 14 days post-injury, although the former chimeric mice with WT macrophages and *Nes-Socs3*^*−/−*^ reactive astrocytes showed significantly ameliorated functional recovery in comparison to WT mice, the latter chimeric mice with *IRF8*^*−/−*^ macrophages and *Nes-Socs3*^*−/−*^ reactive astrocytes exhibited greater deterioration of motor functional recovery in comparison to WT and the former chimeric mice (Fig. 3e). These findings revealed that for motor recovery after SCI, the loss of autonomous macrophage migration due to *IRF8* knockout has a greater impact than the enhancement of reactive astrocyte migration. Regarding their interaction, these findings clarified that macrophage migration plays a leading role in determining their distribution. In addition, Socs3 is a negative feedback factor for Stat3^12,13^, and it is known that Stat3 regulates reactive astrocyte migration via the Rho A small G protein^11^. In this study, the loss of IRF8 in macrophages also altered the astrocyte distribution with or without the expression of *Socs3*. This result also supports the hypothesis that the pathway by which macrophages attract astrocytes is not mediated by Stat3 (Fig. 2i).

### Macrophage-astrocyte interactions via P2Y1 receptors

**Figure 4:**
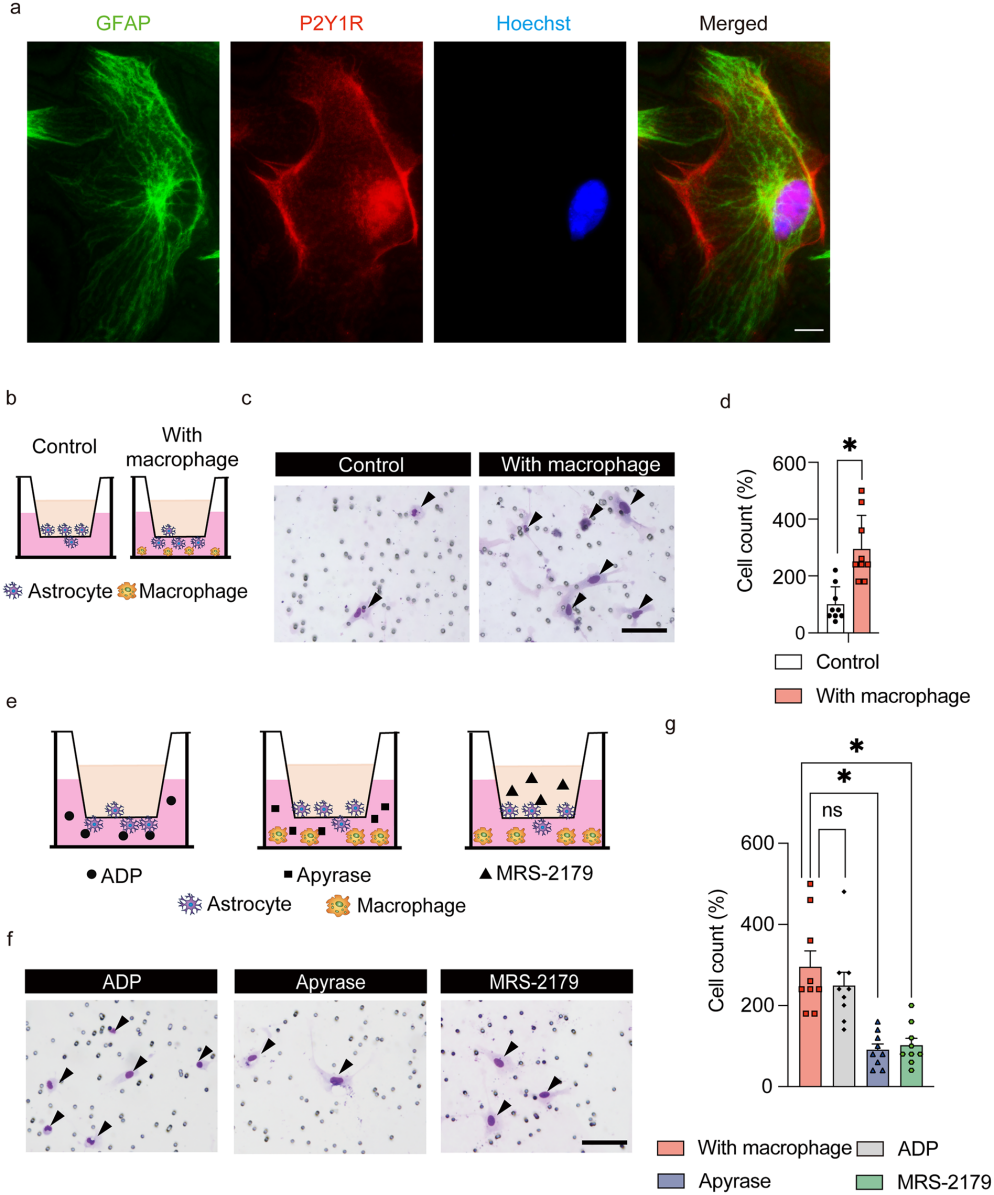
Macrophages attract astrocytes via the P2Y1R. (**a**) The expression of P2Y1R in astrocytes in vitro. Scale bar: 10 μm. (**b**) A schematic illustration of the astrocyte transwell assay with/without macrophages. (**c**) Transwell assay of astrocytes in the control and macrophage groups. Diff-Quik staining images are representative of 2 independent experiments. Scale bar: 100 μm. (**d**) Comparison of the number of migrating cells in the control and macrophage groups (9 sections/3 wells per group). (**e**) A schematic illustration of the transwell assay to reveal the pathways by which macrophages attract astrocytes. (**f**) Transwell assay of astrocytes in the control and macrophage groups. Diff-Quik staining images are representative of 3 independent experiments. Scale bar: 100 μm. (**g**) Comparison of the number of migrating cells between macrophages, without macrophages/with ADP, with macrophages/with apyrase, and with macrophages/with MRS-2179 (9 sections/3 wells per group). *p < 0.05, ordinary one-way ANOVA/unpaired t-test. Error bars indicate the SEM.

To elucidate how macrophages affect the migration of astrocytes, we focused on the P2Y1 receptor (P2Y1R) as a representative receptor in the migration pathways, because P2Y1R was reported to be involved in astrocyte velocity in traumatic brain injury^14^ and to regulate cell migration via the ADP-P2Y1R-MAP/ERK pathway^15^. Therefore, we hypothesized that ATP-derived ADP secreted by macrophages might attract astrocytes via P2Y1R. First, we performed immunostaining to confirm the expression of P2Y1R in the astrocytes of the injured spinal cord. As a result, P2Y1R was expressed in astrocytes in vitro (Fig. 4a). Second, we performed a transwell assay using primary cultured astrocytes. To investigate whether macrophages attract astrocytes, we cocultured the cells with macrophages (Fig. 4b). Coculture with macrophages significantly increased the number of migrating astrocytes (Fig. 4c,d). Next, we performed three experiments to clarify the pathway by which macrophages attract astrocytes (Fig. 4e). First, we evaluated whether ADP attracts astrocytes. The number of migrating astrocytes was significantly increased when a medium with ADP was used, indicating that ADP attracts astrocytes (Fig. 4f,g). Second, to confirm whether the attraction of astrocytes by macrophages was due to the secretion of ADP, we performed coculturing of macrophages and astrocytes with apyrase, an enzyme that degrades ADP (Fig. 4e). The results showed that the number of migrating astrocytes did not increase in the presence of apyrase, indicating that astrocyte migration is regulated by macrophage-derived ADP (Fig. 4f,g). Finally, to determine whether P2Y1R is the receptor on astrocytes on which ADP acts, we cocultured astrocytes and macrophages with MRS-2179, an antagonist of P2Y1R. With MRS-2179, the number of migrating astrocytes did not increase, indicating that astrocytes are attracted to macrophages by the ADP-P2Y1R pathway (Fig. 4f,g). Since ATP secreted from macrophages is degraded to ADP, macrophages attract astrocytes via the ADP-P2Y1R axis, resulting in a shift in astrocyte distribution toward macrophages in the epicenter and astroglial scar formation after SCI.

### Injection of ADP attracts astrocytes to the center of the injured spinal cord

**Figure 5:**
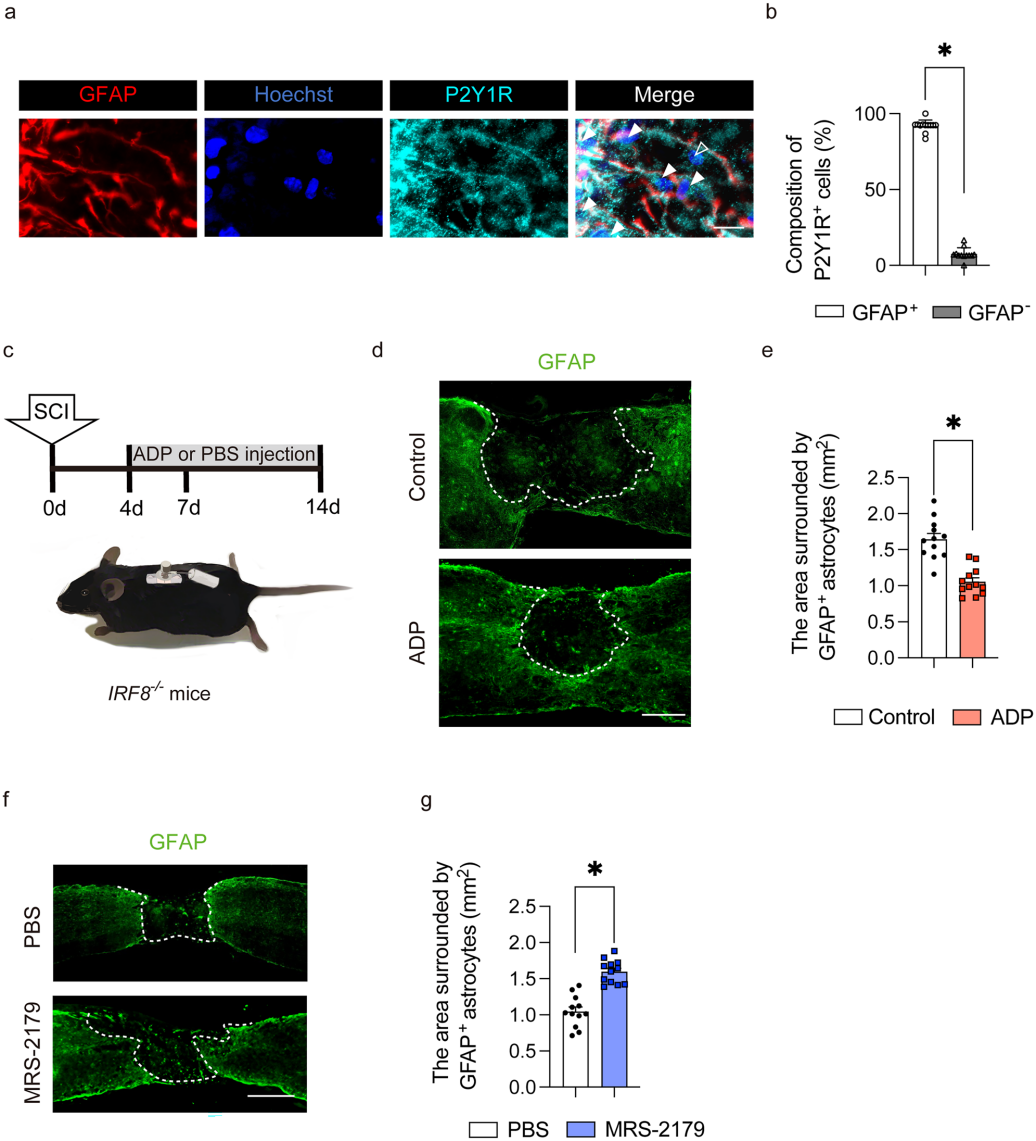
ADP attracts astrocytes in vivo. (**a**) Immunostaining of the injured spinal cord at 7 dpi. Scale bar: 50 μm. (**b**) The quantitative analysis of composition in P2Y1R^+^ cells. There was a significant difference between the GFAP^+^ cells and GFAP^−^ cells. White arrowheads are P2Y1R^+^/GFAP^+^ cells, hollow arrowhead is P2Y1R^+^/GFAP^−^ cell. Scale bar: 50 μm. (**c**) A schematic illustration of continuous intraspinal injection of ADP. (**b**) Immunostaining of the injured spinal cord in the ADP continuous intraspinal injection group and the control group. Scale bar: 500 μm. (**d**) The quantitative analysis of the area surrounded by GFAP-positive cells. There was a significant difference between the ADP continuous intraspinal injection group and the control group (n = 6 per group). (**e**) Immunostaining of the injured spinal cord in the MRS-2179 intraspinal injection group and the PBS intraspinal injection group. Scale bar: 500 μm. (**f**) The quantitative analysis of the area surrounded by GFAP-positive cells. There was a significant difference between the MRS-2179 intraspinal injection group and the PBS intraspinal injection group (n = 6 per group). *p < 0.05, unpaired t-test. Error bars indicate the SEM.

Although P2Y1R could be expressed in many cell types, the majority of P2Y1R-positive cells were astrocytes in the injured area of the spinal cord (Fig. 5a,b). Therefore, we evaluated the administration of ADP to *IRF8*^−/−^ mice, which are known to exhibit extensive macrophage migration after SCI, could shift astrocyte distribution via the ADP-P2Y1R pathway. As ADP is known to be degraded in vivo in a short time, we devised a method of continuous intraspinal injection (Fig. 5c). The continuous intraspinal injection of ADP attracted astrocytes to the center of the SCI and reduced the glial scar area in comparison to controls, which were *IRF8*^*−/−*^ mice treated with PBS (Fig. 5d,e). Next, WT mice were injected with MRS-2179, a P2Y1R antagonist, into the spinal cord during the subacute phase of SCI. In contrast, in mice injected with MRS-2179, astrocytes were not attracted to the center of the injury, and glial scars were enlarged (Fig. 5e,f).

## Discussion

In this study, we showed that macrophages are essential for the shift in astrocyte distribution after SCI using bone marrow chimeric mice. Impairment of macrophage migration led to the widespread distribution of reactive astrocytes. *Socs3*^−/−^ astrocytes, the migration of which is usually promoted and which usually show a narrow distribution, showed widespread distribution with impaired macrophage migration.

These results indicate that macrophages play a leading role over astrocytes in the cell migration of macrophages and astrocytes after SCI. In addition, the coculture of macrophages and astrocytes also showed that macrophages attract astrocytes. Using antagonists, we further elucidated that ADP derived from ATP secreted extracellularly by macrophages attracted astrocytes via P2Y1R. Furthermore, in vivo, the continuous administration of ADP to the center of the injury attracts astrocytes to the center of the injury and reduces the area of the glial scar. These findings indicated that macrophages affect the shift in reactive astrocyte distribution and are important clues to clarifying the pathophysiology of SCI.

Since the mechanisms of glial scar formation are quite complex, we divided glial scar formation into the following stages and focused on each stage: change from naïve astrocytes to reactive astrocytes, astrocytic migration to the injured area, and change from reactive astrocytes to scar-forming astrocytes. Regarding the migration of astrocytes to the injured area, we previously reported that astrocyte migration involves Stat3 and its negative regulator, Socs3, and that the knockout of *Stat3* impedes astrocyte migration^4,11^. Therefore, we initially suspected the involvement of Socs3 in the mechanism through which macrophages attract astrocytes. However, the results of this study showed that Socs3 is not involved in the mechanism through which macrophages attract astrocytes. Therefore, we focused on the P2Y receptor, which has been reported to be involved in multiple cell-to-cell communication pathways in the CNS. For example, microglia, neurons, and astrocytes have been reported to interact via P2Y receptors^14,16^. Although activated macrophages have also been reported to attract astrocytes^17^, the detailed mechanisms through which this occurs remain unclear. In the present study, focusing on P2Y1R, which is expressed in various cells in the CNS, we showed that macrophages significantly affect astrocyte migration, which may occur during the acute to subacute phase, via P2Y1R. While the reduction of macrophages decreases astrocyte scarring^18^, macrophages have also been reported to promote tissue repair^19^. In SCI, macrophages may have a dual role, not only scarring astrocytes via inflammatory cytokines but also attracting astrocytes, and reducing the extent of the scar. Since macrophages comprise the majority of inflammatory cells in the spinal cord scar and astrocytes are the major component of the glial scar, clarifying the interaction between macrophages and astrocytes is essential for elucidating the pathophysiology of SCI.

Although this study showed that macrophages may regulate astrocyte migration as one of the stages in glial scar formation, we have no clear evidence as to whether macrophages are involved in other stages, particularly the change from reactive astrocytes to scar-forming astrocytes. However, it is possible that macrophages may be involved in scarring by secreting collagen or by changing the composition of the extracellular matrix. We have reported that type I collagen triggers the change from reactive astrocytes to scar-forming astrocytes^20^. Macrophages may also contribute to astrocytic scarring because macrophages are known to secrete collagen^21^ and affect the composition of the extracellular matrix by affecting other cells, such as fibroblasts^22^.

In addition, given that the glial scar, once formed in the subacute phase, is maintained into the chronic phase, the macrophage-derived ADP-astrocytic P2Y1R axis may also be involved in the mechanisms of glial scar maintenance^23^. We previously reported that scar-forming astrocytes maintain the glial scar by changing the surrounding naïve astrocytes to scar-forming astrocytes^23^. However, it is not known how astrocytes are recruited to the scar. It has already been reported that astrocytes secrete ATP^24^. Astrocyte-derived ATP is degraded to ADP and may attract other astrocytes. With regard to scar maintenance in the chronic phase, astrocytes may have been recruited to scar tissue via the macrophage- or astrocyte-derived ADP-astrocyte P2Y1R axis.

To date, there have been many reports regarding whether astrocytes migrate after CNS injury. Bardehle et al. reported that GLAST^+^ astrocytes did not migrate. However, there are many different populations of astrocytes^25^. Thus, Nestin^+^ astrocytes, which we defined as reactive astrocytes, and GLAST^+^ astrocytes may be different populations. The difference in the injury models adopted also has a major effect. In the contusion injury model we adopted, the injury area is larger than in the stab injury model, which results in the formation of an astrocyte-deficient space at the center of the injury. This space is filled by astrocytes over time. It is unlikely that this occurs without the migration of astrocytes. Furthermore, Tsai et al. reported that astrocytes are unlikely to migrate outside the originally defined region in the axial direction. However, we showed that labeled astrocytes or transplanted astrocytes migrated in the craniocaudal direction after SCI^4,20^. Therefore, although further discussion and experimentation are needed, in our spinal cord contusion injury model, astrocytes may migrate in a sagittal direction toward the center of injury.

In this study, we showed a mechanism in which macrophages attract astrocytes to the center of injury. Considering that astrocytes are arranged in a row at the outer edge of the scar and are rarely present inside the glial scar, astrocyte migration is not regulated only by the concentration gradient of ADP; some factor inside the scar stops astrocyte migration. However, the mechanism that stops the migration of astrocytes at the glial scar has not been elucidated. For example, PARP1 may be a candidate for the negative regulation of astrocyte migration. Stat3 is known to be essential for astrocyte migration^4^, and PARP1 has been reported to interact directly inhibit Stat3 phosphorylation by causing poly-ADP-ribosylation of Stat3^26^. The expression of PARP1 was upregulated in the spinal cord after SCI in comparison to the naïve spinal cord, as was previously shown in our RNA-seq study^20^. This result is consistent with the hypothesis that PARP1 negatively regulates astrocyte migration in SCI.

In conclusion, we demonstrated that the impairment of macrophage migration led to widespread astrocyte distribution. Macrophages secrete ATP that is degraded to ADP, and astrocytes migrate toward macrophages via the ADP-P2Y1R pathway. Our findings provide deeper insight into the interaction between astrocytes and macrophages and suggest a potential therapeutic target for SCI.

## Methods

### Animals

All study protocols involving mice were approved by the Committee of Ethics on Animal Experimentation of our institution and were conducted in accordance with ARRIVE guidelines (https://arriveguidelines.org) and with the National Institutes of Health guidelines for the care and use of animals. All mice were housed in a temperature and humidity-controlled environment on a 12-h light–dark cycle and had ad libitum access to food and water. *IRF8*^*−/−*^, *Nes-EGFP*^+^, and *Nes*-*EGFP*^+^*-Socs3*^*−/−*^ mice were generated as described previously^4,27^. Bone marrow transplantation was performed as previously described^28^. Eight-week-old female C57BL/6J mice were used as WT mice. Macrophage IRF8-deficient chimeric mice were generated by transferring the bone marrow cells (BMCs) of *IRF8*^*−/−*^ mice into *Nes*-*EGFP*^+^ and *Nes*-*EGFP*^+^-*Socs3*^*−/−*^ recipient mice after irradiation, as previously described^28^.

### Spinal cord injury

Mice were anesthetized with pentobarbital (75 mg/kg intraperitoneally) and subjected to a contusion injury (70 kilodynes) at the 10th thoracic level using an Infinite Horizons Impactor (Precision Systems Instrumentation, Lexington, KY)^29^. After the injury, the overlying muscles were sutured, and the skin was closed with wound clips. During the period of recovery from anesthesia, the animals were placed in a temperature-controlled chamber until thermoregulation was re-established. Motor function was evaluated using a locomotor open-field rating scale, the BMS^30^.

### MRS-2179 injection

A glass tip was inserted at the epicenter of the injured spinal cord, and 2 μl of MRS-2179 (1 mM; Abcam, Cambridge, UK) was injected at 0.5 μl/min using a stereotaxic injector (KDS 310, Muromachi Kikai) at 4 and 7 dpi^31^. Control animals received 2 μl of PBS at 4 and 7 dpi.

### Continuous intraspinal injection of ADP

At 4 dpi, an Alzet Brain Infusion Kit 3 (Alzet 8851; Alzet, USA) was inserted 1.5 mm from the dura mater at the epicenter of the injured spinal cord. An osmotic pump (Alzet 1002; Alzet, USA) filled with 1 mM ADP or PBS and primed for 3 h before surgery was implanted and connected to an Alzet Brain Infusion Kit 3. ADP and PBS were administered continuously from 4 to 14 days after SCI. The delivery rate of the osmotic pump was 0.25 μl/h. ADP was dissolved in PBS and then adjusted to pH 7.4 using 1 mM NaOH.

### Primary astrocyte cultures

Purified primary astrocyte cultures were prepared from C57BL/6J mice, as described previously^4,32^. In brief, after removal of the meninges, on postnatal day 2, mouse brain tissues were minced and incubated in a rocking water bath at 37 °C for 30 min in DMEM (08456-36, Nacalai Tesque Kyoto, Japan) in the presence of 0.25% trypsin (Nacalai Tesque Kyoto, Japan) and 4 mg/ml DNase I (Sigma, Saint Louis, MO). The dissociated cells were triturated with 0.25% FBS and centrifuged at 300×*g* for 3 min. Following dilution with an astrocyte-specific medium: DMEM containing 10% FBS (Life Technologies, Carlsbad, CA) and 1% penicillin–streptomycin (Nacalai Tesque Kyoto, Japan), the cells were plated on a poly-L-lysine-coated T75 flask. After 7 days in a humidified CO_2_ incubator at 37 °C, the T75 flask was set up on an orbital shaker to remove microglia at 180 rpm for 30 min. We added 20 ml of fresh astrocyte culture medium and then shook the flask at 240 rpm for 6 h to remove oligodendrocyte precursor cells. Astrocytes were detached from T75 flasks using 0.25% trypsin and were used for experiments on that day.

### Primary macrophage culture

Purified primary macrophage cultures were prepared from C57BL/6J mice, as described previously^33^. In brief, after removing the muscles and tendons, bone marrow cells were extracted from the femur. Following dilution with macrophage differentiation-specific medium, RPMI 1640 (Nacalai Tesque Kyoto, Japan) containing 10% FBS (Life Technologies, Carlsbad, CA), 1% penicillin–streptomycin (Nacalai Tesque Kyoto, Japan), and 40 ng/ml M-CSF (RSD, Minneapolis, MN 55413), the cells were plated in a poly-l-Lysine-coated T25 flask. After 6 days in a humidified CO_2_ incubator at 37 °C, the macrophages were detached from the T25 flask using EDTA (Nacalai Tesque Kyoto, Japan).

### Quantitative reverse transcription polymerase chain reaction (RT‒PCR)

Total RNA was isolated from the astrocytes obtained from spinal cord tissue using the RNeasy Mini kit (Qiagen, Venlo, the Netherlands). cDNA was synthesized from the total RNA using PrimeScript Reverse Transcriptase (Takara, Tokyo, Japan) according to the manufacturer’s instructions. RT‒qPCR was performed using primers specific to the genes of interest (Table 1) and a SYBR Premix Dimmer-Eraser (RR091A; Takara Bio, Shiga, Japan). Data were normalized to the level of glyceraldehyde-3-phosphate dehydrogenase (GAPDH). Real-time PCR was conducted using a CFX Connect Real-Time PCR Detection System (Bio-Rad, Hercules, CA).

**Table 1:** Primers used for quantitative RT‒PCR.

Gene symbol	Accession number	5ʹ-Forward primer-3ʹ	5ʹ-Reverse Primer-3ʹ
*Slc39a6*	NM_031168.2	TGAAGGCAGCACCAATAGCA	GGCCTGGATGGTGATCATG
*Gapdh*	NM_008084.2	GACTTCAACAGCAACTCCCACTCT	GGTTTCTTACTCCTTGGAGGCCAT

### Histopathological examinations

After animals were anesthetized and transcardially fixed with 4% paraformaldehyde (PFA; Millipore, Burlington, MA), the spinal cord from T7 to T11 was removed, dehydrated, and embedded in an optimal cutting temperature compound (Sakura Finetek Japan, Tokyo, Japan). The sections were mounted on MAS-coated slide glasses (Matsunami Glass, Kishiwada, Japan). Astrocytes cultured in vitro were washed three times with PBS and fixed for 15 min in 4% PFA at room temperature. After washing three times with PBS, these sections and cells were used for immunofluorescence staining. Then, the sections were stained with the following antibodies in a blocking solution overnight at 4 °C: GFAP (1:500; rabbit; Dako, Santa Clara, CA; Z0334), GFAP (1:500; rat; Life Technologies, Carlsbad, CA; 130300), CD68 (1:1000; rat; Bio Rad, Hercules, CA; 94547), and P2Y1R (1:500; rabbit; Allomone Labs, Jerusalem, ISR; APR-021). The primary antibodies were visualized with secondary antibodies conjugated to Alexa 488, 568, 647 (1:1000; Jackson ImmunoResearch, West Grove, PA). The nuclei were visualized with Hoechst 33258 (1:1000; Invitrogen, Waltham, MA). Figures 1b, 2b,g,h and 3c,d were evaluated at the center of injury in 1 section per animal (n = 6). Figure 5b,e,g and Supplementary Fig. S1b were evaluated at the center of injury in 2 sections per animal (n = 6). The area of EGFP^+^ reactive astrocytes in Fig. 2g,h was measured for 20 cells that were randomly extracted from these sections. All images were captured using a BZ-X700 digital microscope system (Keyence, Osaka, Japan).

### Transwell assay

The transwell assay was performed as described previously^34^. Using transwell inserts (No 354480; Corning; NY; 14831), a transwell assay was performed in 5 groups: (1) the control group: primary astrocytes in the inserts, with RPMI 1640 in the well; (2) the macrophage group: primary astrocytes in the inserts, with RPMI 1640 with primary macrophages in the well; (3) the ADP group: primary astrocytes in the inserts, with RPMI 1640 with ADP (100 μM; Oriental Yeast, Tokyo, Japan) in the well; (4) the apyrase group: primary astrocytes in the inserts, with RPMI 1640 with primary macrophages and apyrase (10 U/ml; Sigma, Saint Louis, MO) in the well; and (5) the MRS-2179 group: primary astrocytes treated with MRS-2179 (10 μM; Abcam, Cambridge, UK) in the inserts, with RPMI 1640 with primary macrophages in the well. Each insert was seeded with 5.0 × 10^4^ cells of astrocytes and each well was seeded with 5.0 × 10^4^ cells of macrophages. After incubation for 24 h and staining with Diff-Quick (Sysmex, Kobe, Japan), the number of migrating cells was counted in 9 sections/3 wells per group. The percentage increase in migrating cells compared to the control group was evaluated.

### Statistical analyses

All statistical analyses were performed using the GraphPad Prism software program, version 9.1.2 (GraphPad Software Inc., San Diego, CA.). Figures 2b,g,h, 4d, 5b,e,g and Supplementary Fig. S1b were subjected to an unpaired *t*-test, while Figs. 3c,d and 4g were subjected to an ordinary one-way ANOVA. Figure 3e was subjected to a two-way ANOVA.

### Ethics approval

Ethical approval was obtained from the ethical review committee of Kyushu University Graduate School of Medical Sciences (A25-089-0/A22-336-1) in accordance with the provisions of the institution’s Regulation for Animal Experiments.

## Supplementary Information

Supplementary Legends.

Supplementary Figure S1.

## Data Availability

The datasets generated and/or analyzed in the current study are available upon request from the corresponding author.

## Abbreviations

SCI: Spinal cord injury
*IRF8*: Interferon Regulatory Factor 8
IL-1α: Interleukin-1α
IL-Iβ: Interleukin-1β
IL-6: Interleukin-6
TNF-α: Tumor necrosis factor-α
ATP: Adenosine triphosphate
ADP: Adenosine diphosphate
WT: Wild type
*Nes*: Nestin
*Socs3*: Suppressor of cytokine signaling-3
EGFP: Enhanced green fluorescent protein
DMEM: Dulbecco’s Modified Eagle Medium
RPMI1640: Roswell Park Memorial Institute medium 1640
EDTA: Ethylenediaminetetraacetic acid
GFAP: Glial fibrillary acidic protein
CD68: Cluster of Differentiation 68
*Slc39a6*: Solute Carrier Family 39 Member 6
MAPK: Mitogen-activated protein kinase
ERK: Extracellular signal-regulated kinase
*Stat3*: Signal transducer and activator of transcription 3
