# Supplementary material for: Macrophages play a leading role in determining the direction of astrocytic migration in spinal cord injury via ADP-P2Y1R axis
Source: Sci Rep. 2023 Jul 10;13:11177. doi: 10.1038/s41598-023-38301-8 (PMC10333181; doi:10.1038/s41598-023-38301-8)
Supplement: Supplementary file 1 — Supplementary Legends. [file 41598_2023_38301_MOESM1_ESM.docx]

**Supplementary Figure S1. Composition of Nestin^+^ cells.**

(a) Immunostaining of the injured spinal cord at 7 dpi in *Nes-Cre-EGFP* mice. Scale bar: 50 μm. (b) The quantitative analysis of GFP^+^ cell composition. There was a significant difference between the GFAP^+^ cells and GFAP^-^ cells. White arrowheads indicate GFP^+^/GFAP^+^ cells, hollow arrowhead indicates GFP^+^/GFAP^-^ cell.

**p* < 0.05, unpaired t-test. Error bars indicate the SEM.
