# Supplementary figures and images for: Macrophages play a leading role in determining the direction of astrocytic migration in spinal cord injury via ADP-P2Y1R axis
Source: Sci Rep. 2023 Jul 10;13:11177. doi: 10.1038/s41598-023-38301-8 (PMC10333181; doi:10.1038/s41598-023-38301-8)

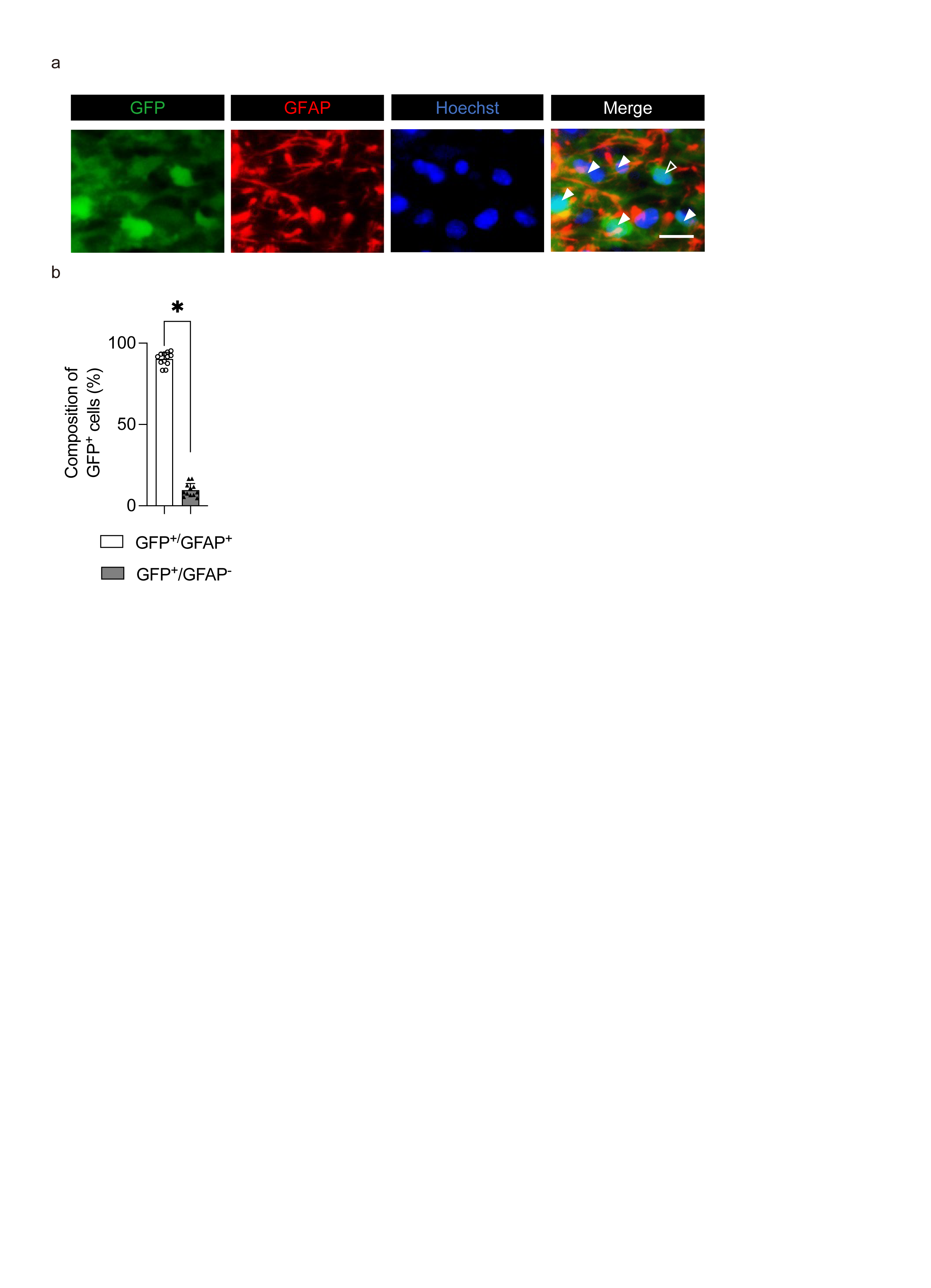

Supplement: Supplementary file 2 — Supplementary Figure S1. [file 41598_2023_38301_MOESM2_ESM.tif]
